# Hydrological Season Can Have Unexpectedly Insignificant Influences on the Elevational Patterns of Functional Diversity of Riverine Macroinvertebrates

**DOI:** 10.3390/biology11020208

**Published:** 2022-01-28

**Authors:** Qingyi Luo, Ming-Chih Chiu, Lu Tan, Qinghua Cai

**Affiliations:** 1 State Key Laboratory of Freshwater Ecology and Biotechnology, Institute of Hydrobiology, Chinese Academy of Sciences, Wuhan 430072, China; luoqingyi@ihb.ac.cn (Q.L.); mingchih.chiu@gmail.com (M.-C.C.); tanlu@ihb.ac.cn (L.T.); 2 College of Advanced Agricultural Sciences, University of Chinese Academy of Sciences, Beijing 100049, China; 3 Department of Entomology, National Chung Hsing University, Taichung 40227, Taiwan; 4 Department of Civil and Environmental Engineering, Ehime University, Matsuyama 790-8577, Japan

**Keywords:** functional diversity, season, riverine macroinvertebrate, elevational variation

## Abstract

**Simple Summary:**

Freshwater ecosystems are increasingly affected by climate dynamics. This study is the first to explore the seasonal effects on the spatial patterns in functional diversity along an elevational gradient. The results showed that the pattern of functional diversity of riverine macroinvertebrates along the elevation gradient was either unimodal or monotonically decreasing. Seasonal changes did not affect the elevational patterns. The findings provide important research and management tools for the temporary effects of river ecosystems.

**Abstract:**

Spatial biodiversity is a key issue in biogeography for the explorations of biological origin and diversification. However, seldom studies have addressed the temporal changes in spatial patterns of biodiversity. We explored the taxonomic and functional diversities of riverine macroinvertebrates in central China, with the elevational gradient, in different seasons in a normal climate year (i.e., no extreme anomalies in the annual precipitation or average annual temperature). The air temperature and streamflow discharge were decreased monotonically with the increase of elevation both in the dry and wet seasons. In addition, the total nitrogen had no significant change with the increase of elevational gradient in the dry season but showed a monotonically decreasing pattern in the wet season. The total phosphorus showed a monotonically decreasing pattern with the elevational gradient in the dry season but had no significant change in the wet season. The spatial pattern of taxonomic diversity of macroinvertebrates along the elevational gradient showed complex patterns, but the functional diversity had either the unimodal or monotonically decreasing pattern. In addition, the functional diversity with the elevational gradient had similar patterns between the dry and wet seasons. Further analysis of the elevational pattern in different seasons is an important basis for understanding the status quo of functional diversity and formulating countermeasures for biodiversity conservation.

## 1. Introduction

How biodiversity changes along spatial gradients is a key issue in biogeography for empirical and theoretical explorations of biological origin and diversification [1,2,3]. The influences of elevation on the structure of biological communities play vital roles in the drivers of global biodiversity [4,5]. Research on the biodiversity along elevational gradients mainly focuses on taxonomic diversity [6]. Nevertheless, a growing number of studies have confirmed that environmental filtering selects biological assemblages with shared biological traits rather than individual species [7]. In terms of ecosystem functions, studies based on biological traits can provide more information than studies merely on species diversity [8]. Using biological traits to represent functional composition and diversity is one of the hotspots in ecological research, e.g., for macroinvertebrates [9]. The functional traits have been proven to serve as a promising proxy of the community or ecosystem functions in response to the various types of disturbances [10,11,12].

The elevational patterns of biodiversity in many biological assemblages, e.g., mammals, plants, fungi, and bacteria, have been examined [13]. For example, rather than showing a universal pattern, e.g., decreased biodiversity with increasing elevations, similar to the latitudinal pattern [14,15], biodiversity elevational patterns are different across regions and taxonomic groups [16,17]. Existing studies have found that the elevational patterns mainly conform to several ones, e.g., the unimodal [18,19] and monotonically decreasing patterns for aquatic invertebrates [20,21]. In harsh and constantly disturbed environments, environmental filtering may be particularly important. It is also possible to limit the functional composition of local communities to taxa with similar biological characteristics of adaptation to the environmental conditions. This adaptation leads to a decrease in functional diversity. On the contrary, under stable conditions, competitive exclusion may reduce the functional similarity among species but increase the functional diversity [22].

By assessing the role of environmental filtration in the changes of community composition, functional diversity becomes significant in understanding the changes of diversity and ecosystem processes [23,24]. A few studies have explored the elevational patterns of functional diversity, and their contradictory findings on the pattern of functional diversity along the elevation gradient have been presented [25,26]. For example, the functional richness of macroinvertebrates in the Rocky Mountains was positively correlated with the size and flow stability of streams along the elevational gradient [27]. Conversely, Thakur & Chawla [28] found that in the high-altitude vegetation of the Western Himalaya, the functional diversity decreased with the increase of elevation. Similarly, de Bello et al. [24] and Gazol et al. [29] observed that the functional diversity of the Alps and Southern Ural decreased with increasing elevation. Zhang et al. [26] found a unimodal pattern with the highest functional diversity near the middle of the gradient. In the dry evergreen Afromontane forest of Hararghe Highland, Southeast Ethiopia, Teshome et al. [23] found that most functional diversity indices decreased with increasing elevation, and their results suggested that elevation is the most critical environmental filter affecting species distribution and community structure.

The ecosystem is dynamic, even at a short-term scale, and the availability of habitats, environmental heterogeneity, and spatial connectivity between the habitats all change over time [30]. The habitat dynamics can result in the temporary varying richness and community composition [31]. Freshwater ecosystems are increasingly affected by the climate dynamics. Research on the comprehensive effects of climatic factors, such as season on freshwater ecosystems, is still in its infancy. The understanding of macroscopic effects on proven indicators, such as macroinvertebrates, can provide important research and management tools of river ecosystems, which can assist in the design and implementation of effective management and conservation strategies. The temporal dynamics of Asian rivers are mostly caused by monsoons; the interannual and annual variations of precipitation and river flow in the basin are significant, and the hydrological situations are complex [32]. Elevational environmental factors (e.g., temperature) have been recognized to affect the composition and diversity of communities distributed along elevations and latitudes [33,34]. However, there is still a lack of research on the seasonal effects on the elevational patterns of functional diversity, especially in freshwater ecosystems. 

Our study is the first to explore the seasonal influences on the changes in functional diversity along elevational gradients. The study presented a mechanistic highlight on the influence of climatic factors on the elevational patterns of functional diversity. Macroinvertebrates have important ecological functions in river ecosystems [35,36]. They are sensitive to natural environmental fluctuations and human disturbances and can comprehensively reflect the dynamic changes of aquatic ecosystems at spatiotemporal scales [37]. Our aims are as follows: (1) to explore the spatial patterns of functional diversity of riverine macroinvertebrates along the elevational gradient; (2) to assess the importance of seasons in the elevational patterns of functional diversity. 

## 2. Materials and Methods

The whole year was divided into two periods, according to the temporal distribution of rainfall and hydrological change (i.e., dry and wet seasons) in our study area. We calculated the functional diversity indices of the sampling communities in different seasons. Finally, we assessed the elevational patterns of functional diversity in the different seasons. The elevational gradient integrates a variety of environmental factors. In this study, four environmental factors (air temperature, flow, total nitrogen, and total phosphorus) were measured or collected to explore the driving factors behind the elevational patterns of biodiversity in different seasons.

### 2.1. Study Area

**Figure 1:**
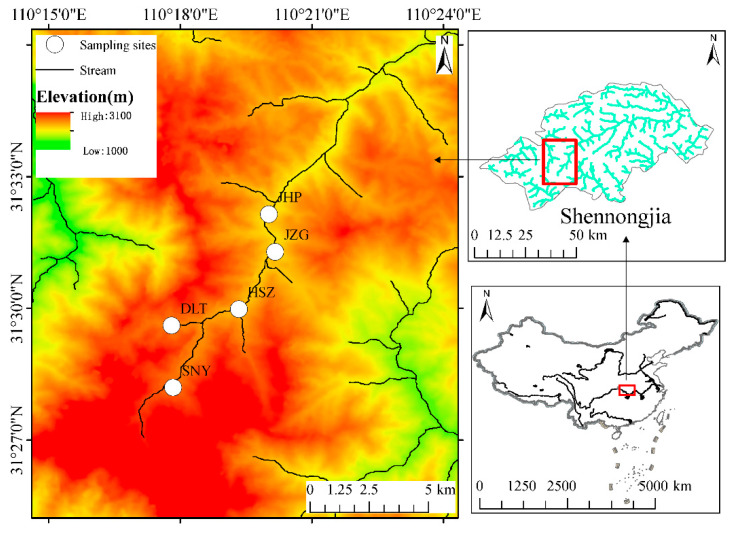
Distribution of the sampling sites in the Shennongjia area of central China.

We conducted this study in Shennongjia, which is a national nature reserve in Hubei Province, China. Shennongjia’s highest peak, Shennongding, is 3105 m above sea level, which also is the highest in central China, with the height of the lowest elevation peak being 420 m [38]. The unique geographical location and complex terrain, as well as the highly dendritic features of the water system, allow this region to be one of the three areas with the highest biodiversity in China. Therefore, Shennongjia Mountain presents an ideal location to study the elevational patterns of biodiversity [39]. In addition, Shennongjia Nature Reserve is located in the north subtropical monsoon climate zone, showing a vertical zonal climate from the foot to the top of the mountain. The annual average precipitation is from 800 to 2500 mm, and there are obvious seasonal changes in precipitation, with the most rainfall in summer (June, July, and August) and the least rainfall in winter (December, January, and February). The annual precipitation is concentrated in July, August, and September, accounting for about 40% to 45% of the annual precipitation. There are also significant differences in precipitation at different elevations. The higher the elevation, the greater the annual precipitation. The highest precipitation in the low altitude areas occurs in May, and the highest precipitation in the high mountain areas occurs in September [40,41]. Under the influence of monsoons, the regional climate is characterized by dry (January–March and October–December) and wet (April–September) seasons. This study conducted monthly sampling at five sites from July 2011 to June 2012 (Figure 1). During our study period, the sampling reaches did not dry up at the five sites. Based on the annual average temperature and annual precipitation data of the Shennongjia area from 1982 to 2021 from Worldclim (http://www.worldclim.com/) (accessed on 18 January 2022) (Figure S1), the sampling period was a normal climate year. The annual average temperature and annual precipitation were in the normal range, and there were no abnormal or extreme values.

### 2.2. Macroinvertebrates’ Sampling and Identification

The macroinvertebrates were collected using Surber net (30 × 30 cm^2^, mesh size = 500 μm). Each sampling site was set with five replicates. The macroinvertebrates were preserved in 95% alcohol for sorting and identification in the laboratory. Most macroinvertebrates were identified to the generic levels, but Chironomidae was identified to subfamily levels. Other taxa, such as Oligochaeta and Turbellaria, were identified to the class levels.

### 2.3. Environmental Variables

Stream width was measured along three representative cross transects. Depth and velocity were measured at 50 cm intervals across a transect using a digital water velocity meter (Global Water FP201, Global Water Instrumentation, Sacramento, CA, USA). The mean values of stream width, depth, and flow velocity were used to calculate streamflow discharge. A 350 mL water sample was collected simultaneously with the macroinvertebrate sampling and preserved by adding sulfuric acid to regulate pH < 2 in the field. The concentrations of total phosphorus (TP) and total nitrogen (TN) were measured in the laboratory using a segmented flow analyzer (Skalar San++ Skalar, Delft, The Netherlands). Monthly average data of air temperature from July 2011 to June 2012 were downloaded from Worldclim (http://www.worldclim.com/) (accessed on 15 October 2021).

### 2.4. Biodiversity Indices

Two taxonomic diversities (the species richness index and Shannon–-Wiener index) were calculated using the vegan package [42] in the R3.6.1 software [43]. Species abundance data were used to calculate the Shannon–Wiener index. The calculation of functional diversity was based on the description of Poff et al. [44] by selecting 20 functional traits. These traits are sensitive to environmental changes (including adult flying strength, swimming ability, attachment, armoring, shape, respiration, rheophily, trophic habit, etc.). Trait data for each taxon were obtained from various sources, including information published in the literature [44,45,46]. These characteristics of the individual taxa were determined according to the description of the specimen or classification data. According to Colzani et al. [47], discrete numbers (i.e., 1, 2, and 3) were used to assign ordered characters, 0 and 1 were used to assign binary characters, and factor characters were retained as the original character description for the subsequent analysis (Table S1). 

The functional richness (FRic) [48], functional evenness (FEve), and functional divergence (FDiv) are calculated as follows:

(1) $$\mathrm{FRic}=\frac{SF_{ci}}{R_{c}}$$

where $SF_{ci}$ is the niche space occupied by species in the community *i*, and $R_{c}\;$ is the absolute value range of character *c.*

(2) $$\mathrm{FEve}=\frac{\sum_{i=1}^{s-1}cmin\left(PEW_{i}\frac{1}{S-1}\right)-\frac{1}{S-1}}{1-\frac{1}{S-1}}\;$$

(3) $$\mathrm{FDiv}=2/\pi \;arctan\left[5\times \overset{N}{\underset{i=1}{\sum }}\left[{\left(lnC_{i}-\left(\bar{lnx}\right)\;\right)}^{2}\times A_{i}\right]\right]$$

where $C_{i}\;$ is the value of the *i* functional trait, $A_{i}$ is the relative abundance of the *i* functional character, and $\bar{lnx}$ is the weighted average of the natural logarithm of the characteristic values of the taxon.

Rao’s Quadratic (RaoQ) index combines the relative abundance of taxon and paired functional differences between taxon, expressing the average difference in traits between any two randomly selected individuals [49]. RaoQ index can be regarded as the extension of the Simpson diversity index in the functional diversity dimension [50]. When there are no shared traits among all species, the value of the Simpson diversity index represents the maximum value that the RaoQ index can reach [51]. The above functional diversity index was calculated by using the dbFD function of the R3.6.1 software with the FD function package [52]. 

### 2.5. Generalized Additive Model

Using the generalized additive model (GAM), we studied the changes of taxonomical or functional diversity with the elevational gradient and seasonal influences on the elevational patterns of functional diversity. In addition to the seasons and elevations, the diversity of macroinvertebrates is affected by the combined effects of physical and chemical factors in the water body, and we also assessed the elevational patterns of environmental factors in different seasons. The dynamic relationship between them is complex, nonlinear, and uncertain. Therefore, GAM can reflect the essential connection between the response and explanatory variables rather than the parameter form, and it is suitable for explaining nonlinear or nonmonotonic data analysis [53]. In this study, the ‘mgcv’ package [54] of the R Programming Language platform was used to analyze the generalized additive model. In the GAM, we used the different distributions to the environmental factors and taxonomic and functional diversity indices (Table 1).

## 3. Results

### 3.1. Assemblages of Macroinvertebrates 

A total of 88 taxa of macroinvertebrates were identified, belonging to 43 families and 84 genera. Among them, *Alloperla* sp. (Insecta: Plecoptera), *Baties* sp. (Insecta: Ephemeroptera), *Heptagenia* sp. (Insecta: Ephemeroptera), *Chironomus* sp. (Insecta: Diptera), *Drunella* sp. (Insecta: Ephemeroptera), *Nemoura* sp. (Insecta: Plecoptera), *Epeorus* sp. (Insecta: Ephemeroptera), *Iron* sp. (Insecta: Ephemeroptera) were the dominant taxa, with relative abundance of 13.99%, 12.43%, 11.29%, 9.47%, 8.79%, 7.69%, 7.51%, and 5.52%, respectively (Table S2).

### 3.2. Environmental Factors along the Elevational Gradient

**Figure 2:**
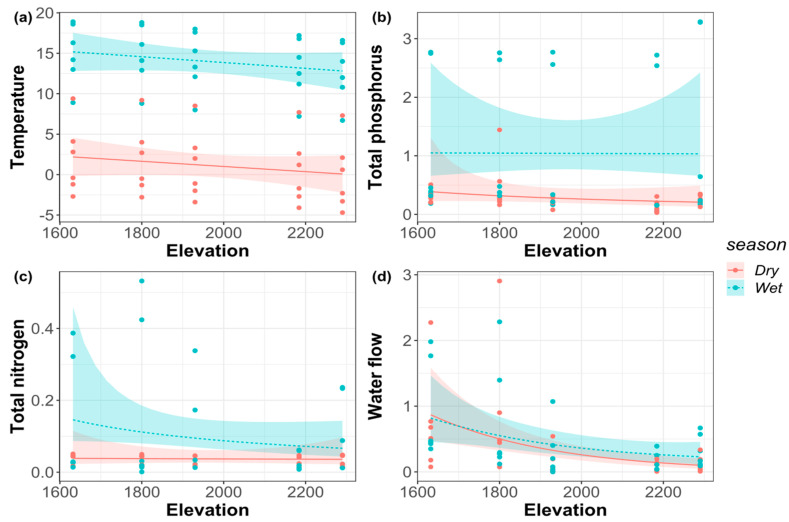
(**a**) Air temperature; (**b**) total phosphorus (TP); (**c**) total nitrogen (TN); (**d**) water flow along the elevational gradient in the dry and wet seasons.

In both the dry and wet seasons, elevation had a significant effect on discharge (*p* < 0.05), while elevation had no significant effect on total nitrogen, total phosphorus, and air temperature (Table 2). The air temperature and streamflow discharge decreased monotonously with the increase of elevation both in the dry and wet seasons (Figure 2). In addition, the total nitrogen had no significant change with the increase of elevational gradient in the dry season but showed a monotonically decreasing pattern in the wet season. The total phosphorus showed a monotonically decreasing pattern with the elevational gradient in the dry season but had no significant change in the wet season.

### 3.3. Taxonomic Diversity along the Elevational Gradient 

According to the results of GAM in Table 3, in the dry season, elevation had a significant effect (*p* < 0.05) on Shannon–Wiener index. However, elevation had no significant effect (*p* > 0.05) on the species richness index. In the wet season, elevation had no significant effect on Shannon–Wiener index and species richness index.

**Figure 3:**
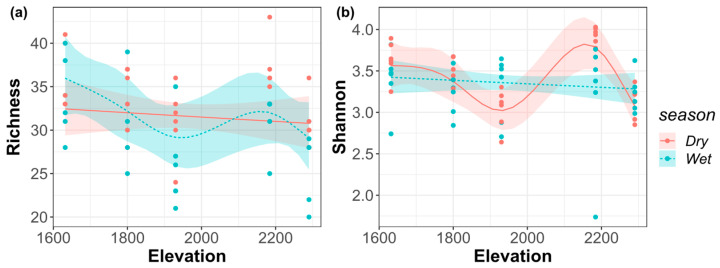
(**a**) Species richness index; (**b**) Shannon–Wiener index of the riverine macroinvertebrate communities along the elevational gradient in the dry and wet seasons.

Species richness was decreased monotonically with the elevational gradient in the dry season; in the wet season, the general fluctuation trend was monotonously decreasing until around 1900 m, and then the richness achieved a peak at around 2200 m (Figure 3a). In the dry season, the overall fluctuation of Shannon–Wiener index showed a monotonous decrease until around 1900 m, which was followed by a peak at around 2200 m; in the wet season, it shows a monotonously decreasing pattern with the elevational gradient (Figure 3b).

### 3.4. Functional Diversity along the Elevational Gradient 

According to the results of GAM in Table 4, in the dry season, elevation had a significant effect (*p* < 0.05) on functional divergence (FDiv), Rao’s Quadratic (RaoQ) index, and functional dispersion (FDis). However, elevation had no significant effect (*p* > 0.05) on functional richness (FRic) and functional evenness (FEve). In the wet season, elevation had a significant effect (all but Feve were significant at *p* < 0.05) on the FDiv, FRic, RaoQ, and FDis but no significant effect on FEve.

**Figure 4:**
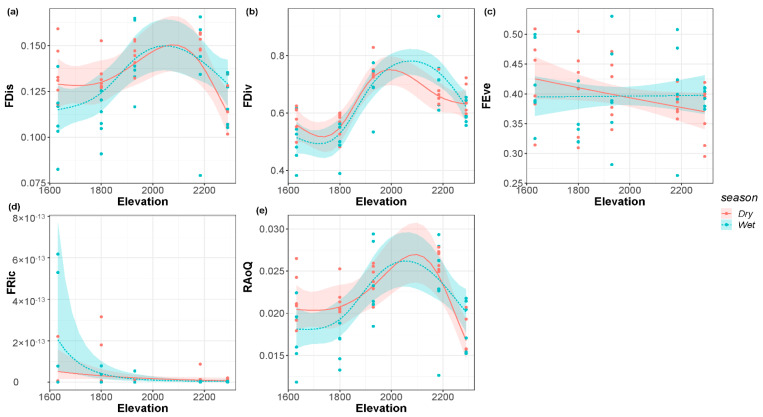
(**a**) Function dispersion; (**b**) functional divergence; (**c**) functional evenness; (**d**) functional richness; (**e**) Rao’s Quadratic of the riverine macroinvertebrate communities along the elevational gradient in the dry and wet seasons.

FDis, FDiv, and RaoQ all presented a single peak pattern in the dry and wet seasons (Figure 4a,b,e, respectively). The FDis increased with the elevational gradient and then reached a summit at an elevation of about 2100 m. FDiv also showed the similar pattern with the change of elevation and reached its peak at an elevation of 2000 m in the dry season, while the peak at an elevation of 2100 m in the wet season was higher than that in the dry season. The peak of RaoQ in the wet season was about 2000 m above sea level. The elevation of the peak in the dry season was higher than that in the dry season, which is about 2100 m. FEve decreased monotonically with the elevational gradient in the dry season (Figure 4c) but did not change significantly in the wet season. FRic showed a monotonically decreasing pattern with the elevational gradient in both the dry and wet seasons (Figure 4d).

## 4. Discussion

We believe that the current study is the first to explore seasonal influences on the elevational patterns of functional diversity. The spatial pattern of taxonomic diversity of the macroinvertebrates in Shennongjia along the elevational gradient showed a general decreasing distribution, and the spatial pattern of functional diversity mainly conformed to the unimodal pattern or monotonically decreasing distribution. Moreover, the regularity of taxonomic diversity was not as clear as that of functional diversity. The season had no significant effect on the distribution pattern of functional diversity of the macroinvertebrates. The four indices (FDis, FDiv, FRic, and RaoQ) varied with the elevational gradient both in the dry and wet seasons. However, the seasons, divided according to rainfall variations, did not affect the elevational patterns of these functional diversity indices of the communities of macroinvertebrates. The streamflow discharge was most significantly affected by the change of elevational gradient. Total nitrogen (TN) and total phosphorus (TP) did not vary significantly along the elevation gradient in the dry season, and both values were lower in the dry season than in the wet season.

### 4.1. Elevational Patterns of Functional Diversity 

Previous studies have found that the elevational changes in the taxonomic and functional diversities of macroinvertebrates mainly conform to the unimodal [19,55] and monotonically decreasing patterns [20,21]. Our results showed that the elevational patterns of functional diversity of macroinvertebrates were in line with the general hypothesis. However, taxonomic diversities usually showed unexpectedly high values at the low elevations. With the general mechanisms, the elevational pattern should be the unimodal or monotonically decreasing pattern. Based on the monotonically decreased pattern, the unimodal pattern is additionally formed with the diversity reduction caused by human disturbances in areas at lower elevations. In our cases, the functional diversity was decreased, but the number of species with similar functions was increased at low elevations. Based on biological traits, functional diversity describes community composition, species’ demands and responses to ecological processes, and their ecological functions [56]. The results of this study also showed that functional diversity is more in line with the response of species to the ecological impacts, but taxonomic diversity could not reflect it in our area.

In harsh and constantly disturbed environments, environmental filtering may be particularly important and may limit the functional composition of local communities to taxa with similar biological characteristics, thereby reducing the functional diversity. On the contrary, the competitive exclusion may reduce the functional similarity and then increase the functional diversity under stable conditions [22]. With the rising elevation, the natural environments may limit the distribution of the most generalist species due to low temperatures and limited food resources [57]. The environmental filtration continues to increase, resulting in a decrease in the niche suitable for species survival, which might be a mechanism of the monotonically decreasing patterns in our study.

This harsh environment also restricts human activities to areas with lower elevation where human disturbances result in changes in riparian vegetation, river morphology, and the river environment, such as water quality river sediment composition [58]. These changes have harmful effects on the macroinvertebrates. Therefore, areas with higher elevation have milder physical environments (e.g., dense vegetation compared with areas at high elevation), and there are almost no human influences. Thus, there are more species at mid-elevations than at higher and lower elevations. In terms of the mid-domain effects, the range of species is randomly distributed in the geographic areas where species richness has the largest overlap in the center of the domain [59]. This effect may be another mechanism of the unimodal pattern of large invertebrate diversity [55,57]. The present study confirms that even in mountains with lower elevations, elevation had a strong impact on community structure, ecosystem function, and environmental characteristics.

### 4.2. Seasonal Influences

Under the influence of the monsoonal climate, there are seasonal fluctuations in rainfall and hydrology in Asia (or areas under Mediterranean climate conditions). The elevational gradient combines the shifted effects of various environmental factors (such as temperature, precipitation, and light) [60] and, thus, is an important factor, affecting the species composition, the construction of the biome, and spatial patterns of diversity [2]. With the increase of elevation, air temperature is expected to decline, while, at the same time, regulate the water temperature and increase the snow cover [61]. In particular, when the relevant environmental gradients and ecological characteristics are concerned, latitude and elevational gradients can be regarded as analogs [62,63]. Different elevations and seasons receive varying amounts of solar radiation and precipitation [64,65]. In turn, this directly affects important instream variables, such as water temperature and streamflow [64,66]. By analyzing the elevational patterns of environmental factors in different seasons, the streamflow discharge was most significantly affected by the change of elevational gradient in our study.

Thakur & Chawla’s [28] results showed that the gradients of aridity (aspect) and decreasing temperature (elevation) on species distribution and functional diversity suggest that the functioning of high altitude communities will be affected in the future. This highlights the function and stability of plant communities at high elevations that are more vulnerable to the effects of climate change. 

Our study showed that seasonal influences did not affect the elevational patterns of the functional diversity of macroinvertebrates. The possible reasons include the following: (1) The study system is a river with gravel sediments. During periods of low flow, aquatic insects can still find suitable refuges to survive; (2) The effect of temperature on the macroinvertebrates masked the effects of high flow in summer. The existing temporal and spatial patterns of water resources cause a high degree of heterogeneity of river habitats, but if the flow is reduced, the physical and chemical characteristics of the river (such as water temperature, nutrients, suspended solids, etc.) will tend to be similar [67]. Total nitrogen and total phosphorus did not vary significantly along the elevation gradient during the dry season. Total nitrogen and total phosphorus levels reflect the nutrient inputs to the river ecosystem and, particularly, affect the photosynthetic productivity of the watershed ecosystem.

### 4.3. Functional Diversity Indices

The study showed that the five indices had different patterns in response to elevational gradients. In extreme cases, the northward movement of the subtropical monsoonal climate may cause some tributaries to dry up or form seasonal rivers, which will strongly affect the biodiversity of the Shennongjia area [68]. However, there was an impact on functional uniformity, which was manifested as the declining pattern with increasing elevation in the dry season but no obvious change in the wet season. The possible reasons include the following: in the dry season, when rainfall and streamflow are small, the impact of the monsoonal climate reduces the types of functional characteristics of the species. The dry season (January–March and October–December) has a lower average temperature compared to the wet season (April–September). Cold-tolerant species are more dominant in the dry season. The reduction in riparian vegetation during the dry season also resulted in limited nutrient input to the river. As can be seen from the results of this study, in the dry season, total nitrogen and total phosphorus are significantly lower than the wet season values. In addition, the life history patterns of macroinvertebrates are important [69]. High temperatures in the wet season, especially in summer, resulted in fast macroinvertebrate growth. In addition, Ephemeroptera is most dominant macroinvertebrates in the Shennongjia area and spawns during the wet season. The species characteristics resulted in a large increase in Ephemeroptera numbers during this period compared to other populations. Thus, changes in the community structure over time affect functional uniformity [70,71]. 

In the wet season, rainfall was sufficient and the most important streamflow has mostly met the hydrological requirements of the macroinvertebrates in our system. Temperature and light were also more suitable in this area. Abundant macroinvertebrate species might contribute to the higher biological diversity. In turn, various functional characteristics of the macroinvertebrates are also abundant. Therefore, in the wet season, the functional evenness was not affected significantly with the elevational gradient. Community structuring is mainly caused by two potential mechanisms, i.e., environmental filtering and restricted similarity [7]. Environmental filtering would select species with the shared traits to coexist in similar environments, while restricting similarity, achieved through their interactions (i.e., through competition) among species, might decrease the coexistence of species. 

### 4.4. Unconsidered Aspects

This study explored the influence of the season on the functional diversity of macroinvertebrates for the first time and achieves a breakthrough from zero to one, but it is still not perfect, and the unconsidered aspects need to be solved in future research. The elevational gradient of the Shennongjia area is 420~3105 m. Because there is no stream distribution at certain elevations and the terrain is complex and difficult to sample, this study only explored streams between 1500 and 2300 m. In the follow-up study, more streams with different elevations or other areas with more suitable elevations can be considered for future studies. In addition, future efforts can address multiple years to accurately reveal the potential mechanism of seasons on the elevational patterns of functional diversity. The variation of species diversity on broad elevation gradients largely depends on the sampling effect [72]. In addition, although the ecological environment is an important driving factor for the elevation pattern of species richness, the history of lineage and the internal diversity in the tree of life is the basis for understanding the diversity of the elevation pattern and the shape of elevation slope. In addition to exploring the elevation pattern of functional diversity, phylogenetic diversity can also be considered in the future exploration direction.

The density and richness of macroinvertebrates are mainly determined by the conditions of hatching, growth, survival rate, emergence, and symbiotic community under the influences of multiple environmental factors, e.g., heat [66,73]. For example, with the increase of temperature, the number of predatory fish and other organisms feeding on macroinvertebrates will increase [74], while the decomposition rate of litters will increase [75] and, thus, reduce the number of macroinvertebrates [68]. Future studies, such as comparative studies covering multiple rivers and mountains, are needed to clarify the underlying mechanisms that drive the spatial pattern of biota elevation in alpine rivers. 

## 5. Conclusions

Understanding the underlying mechanisms of the spatial changes of biodiversity patterns can provide the scientific basis for biological monitoring, protection, and restoration [76,77]. This study analyzed the patterns of functional diversity of macroinvertebrate communities with the elevational gradient under the seasonal influences. The results showed that the riverine communities at an elevation of about 2100 m might make the best use of the niche space and resources in the Shennongjia area of central China in the normal climate year. The seasonal influences did not affect the elevational patterns of macroinvertebrates. Multiple ecological processes and environmental conditions (such as environmental filtration, diffusion, and species interactions) [78,79] are possible explanatory factors for affecting the elevational patterns. Analysis of the elevational patterns in different seasons can confirm the importance of the temporal factor, especially when seasonal influences are generally considered to be one of the key drivers. This step is a critical basis for understanding the status of functional diversity and formulating strategies for biodiversity conservations.

## Figures and Tables

**Table 1:** Distributions used for the biodiversity indices and environmental factors in the generalized additive models.

Variable	Distribution	Data Type and Range
Air temperature	Gaussian	−INF~INF
Total phosphorus (TP); Total nitrogen (TN)	Gamma	0~INF
Water flow; Functional richness (FRic); Functional evenness (FEve); Shannon–Wiener index	Tweedie	≥0
Functional divergence (FDiv)	Quasi-binomial	0~1
Functional dispersion (FDis); Rao’s Quadratic (RaoQ)	Beta	0~1
Species richness	Poisson	Positive integers

Note: INF means infinity.

**Table 2:** GAM results of the relationships between environmental factors and elevational and seasonal predictor variables.

Season	Temperature		Total Phosphorus (TP)		Total Nitrogen (TN)		Flow	
	*p*	Adjusted R^2^	*p*	Adjusted R^2^	*p*	Adjusted R^2^	*p*	Adjusted R^2^
Dry season	0.28	0.74	0.24	0.11	0.89	0.06	<0.01 *	0.13
Wet season	0.22		0.98		0.13		0.03 *	

Note: * means significant difference (*p* < 0.05).

**Table 3:** GAM results of the relationships between the taxonomic diversities and elevational and seasonal predictor variables.

Season	Richness		Shannon	
	*p*	Adjusted R^2^	*P*	Adjusted R^2^
Dry season	0.55	0.11	<0.01 *	0.29
Wet season	0.14		0.43	

Note: * means significant difference (*p* < 0.05).

**Table 4:** GAM results of the relationships between the functional diversities and elevational and seasonal predictor variables.

Season	FDiv		FRic		RaoQ		FEve		FDis	
	*p*	Adjusted R^2^	*p*	Adjusted R^2^	*p*	Adjusted R^2^	*p*	Adjusted R^2^	*p*	Adjusted R^2^
Dry season	<0.01 *	0.58	0.06	0.21	<0.01 *	0.39	0.07	0.02	<0.01 *	0.30
Wet season	<0.01 *		<0.01 *		<0.01 *		0.93		<0.01 *	

Note: * means significant difference (*p* < 0.05). FDiv: functional divergence; FRic: functional richness; RaoQ: Rao’s Quadratic; FEve: functional evenness; FDis: functional dispersion.

## Data Availability

The data presented in this study are available from the corresponding author, upon reasonable request.

## Supplementary Materials

The following are available online at https://www.mdpi.com/article/10.3390/biology11020208/s1, Figure S1: Annual average temperature and annual precipitation from 1982 to 2021 in Shennongjia Area, Table S1: classification table of functional traits of riverine macroinvertebrates communities in Shennongjia, Table S2: the dominant taxa of riverine macroinvertebrates communities in Shennongjia.
